# Supplementary material for: Basal constriction during midbrain–hindbrain boundary morphogenesis is mediated by Wnt5b and focal adhesion kinase
Source: Biol Open. 2018 Oct 25;7(11):bio034520. doi: 10.1242/bio.034520 (PMC6262868; doi:10.1242/bio.034520)
Supplement: Supplementary information [file biolopen-7-034520-s1.pdf]

SUPPLEMENTARY MATERIAL

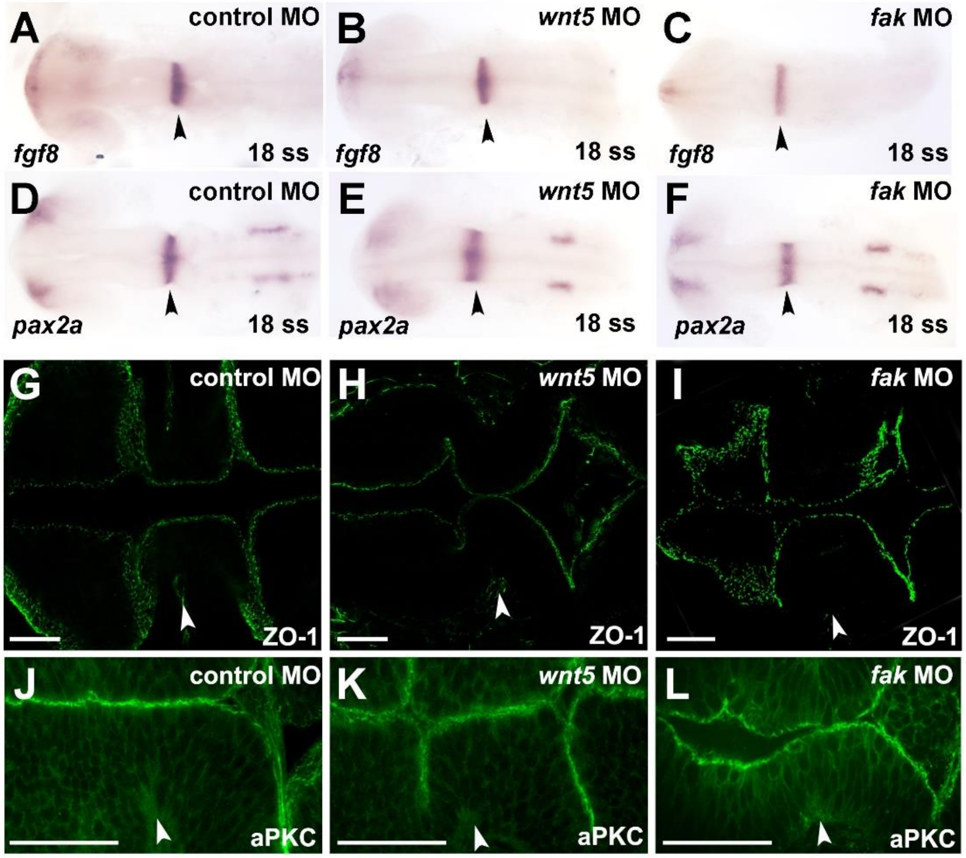

Fig. S1. Tissue patterning and polarity are retained in wnt5b and fak morphants. (A-F) *in situ* hybridization patterns for *fgf8* and *pax2a* expression in control MO (A,D), *wnt5b* MO (B,E), and *fak* MO (C,F) injected embryos at 18 ss. Expression patterns are normal in both *wnt5b* and *fak* morphants. RNA probes containing digoxigenin-11-UTP were synthesized from linearized plasmid DNA for *pax2.1* (Krauss et al., 1991), and *fgf8* (Reifers et al., 1998) as previously described (Harland, 1991). Standard methods for hybridization and for single color labeling were used as described (Sagerstrom et al., 1996). After staining, embryos were de-yolked, flat-mounted in glycerol and imaged with a Nikon compound microscope. (G-L) Immunohistochemistry staining for the apical junction marker ZO-1 (G-I) and the cell polarity maker aPKC (J-L) in control MO (G,J), *wnt5b* MO (H.K), and *fak* MO (I,L) injected embryos at prim-6. Apical localization of ZO-1 appears normal in each condition and indicates establishment of apical junctions. aPKC is also normal in each condition indicating that the cells have established cell polarity. For immunostaining experiments, embryos were fixed in 4% paraformaldehyde or Dent's (70% methanol: 30% DMSO) for ZO-1. Embryos were blocked in 2% normal goat serum, 1% BSA, and 0.1% Triton-X100 in PBT; incubated overnight at 4°C in primary antibody (anti-aPKC (C-20), SC-216, Santa Cruz Biotechnology, 1:1000; anti-ZO1, 33-9100, Invitrogen, 1:200); then incubated in secondary antibody (goat anti-rabbit or anti- mouse IgG conjugated with Alexa Fluor 488, Invitrogen, 1:500). Arrowheads indicate MHBC. Scale bars: G-I, 20 μm; J-L, 35 μm.

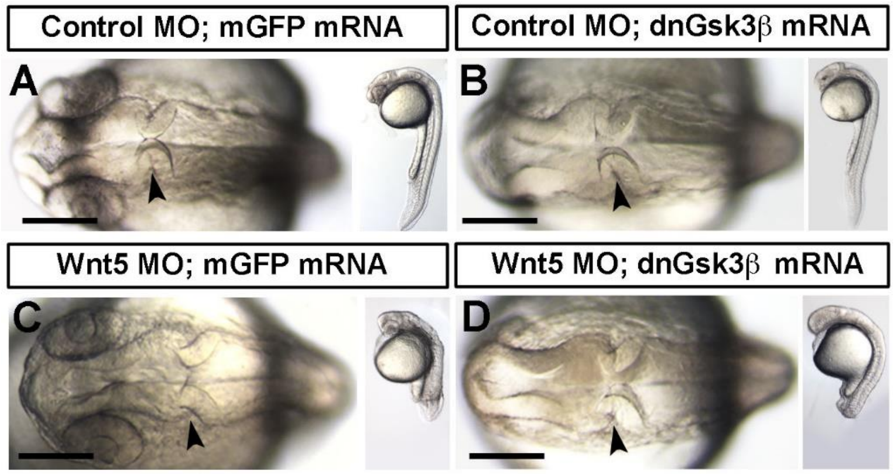

Fig. S2. Gross morphology images of dnGsk3β and wild-type Gsk3β overexpression phenotypes and dnGsk3β rescue of wnt5b morphants. (A-D) Brightfield dorsal and lateral images of control MO (A,B) and *wnt5b* MO (C,D) injected embryos, co-injected with mGFP (A,C), or *dnGsk3*β (B,D) mRNA. (A) Control morphants co-injected with mGFP mRNA demonstrating a normal MHBC basal constriction phenotype. (B) Control morphants co-injected with *dnGsk3*β mRNA exhibit an eyeless phenotype, but undergo basal constriction normally with this concentration of dnGsk3β (*n*=9). (C) *Wnt5b* morphants co-injected with control mRNA exhibit abnormal MHBC morphogenesis, tail defects, and fail to undergo basal constriction (*n*=6). (D) *Wnt5b* morphants co-injected with dnGsk3β mRNA exhibit a loss of eyes and tail defects, but the gross morphology of basal constriction is rescued and occurs normally (*n*=6). Arrowheads indicate the MHBC. Scale bars: 100μm.

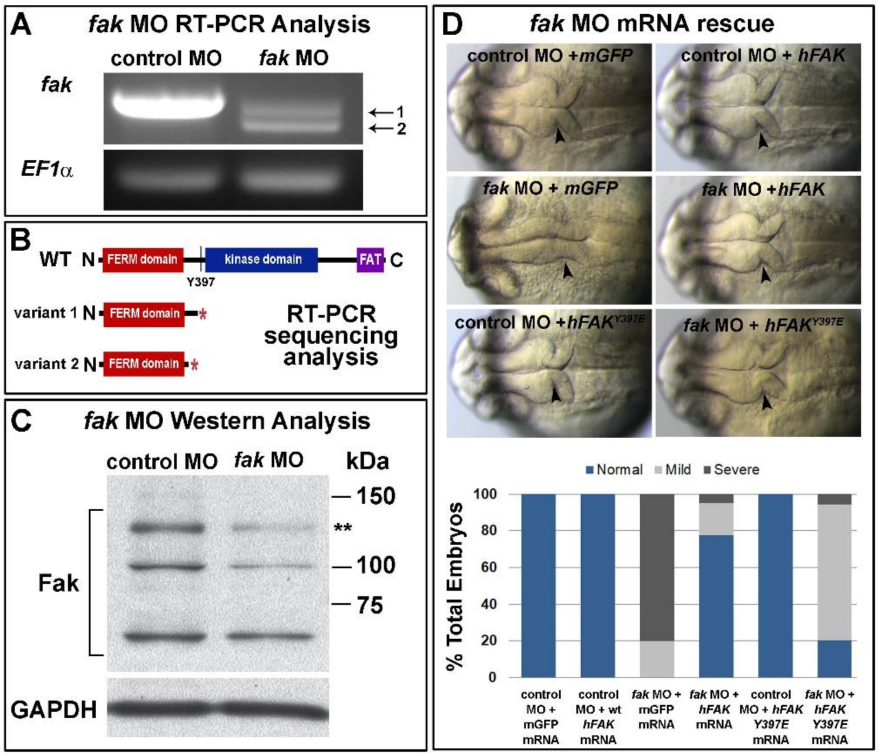

Fig. S3. fak MO efficacy and specificity. (A) RT-PCR for control MO and *fak* MO injected embryos. Whole tissue lysate was analyzed. *fak* MO injection resulted in 2 abnormal mRNA products (arrows 1 and 2). *EF1α* was used as an RT-PCR control. Primers used for RT-PCR: *fak* exon 11 forward 5-CACCTTGCCAACTTCACTCA-3; *fak* exon 22 reverse 5-GTGAATCGTGGGCGTTTACT-3; *EF1α* forward, 5-GATGCACCACGAGTCTCTGA-3; and *EF1α* reverse, 5-TGATGACCTGAGCGTTGAAG-3. *fak* RT-PCR products were cloned into pGEM using the pGEM T-Easy Vector System Kit (Promega) and sequenced. (B) Sequence analysis of the two RT-PCR product variants from (A) resulted in the detection of two truncated mRNA amplicons caused by partial or complete exon deletion, each resulting in early stop codons as indicated in the diagram. Both truncations eliminate the autophosphorylation site Y397. (C) Western Blot analysis of *fak* morphant lysate demonstrating downregulation of full length Fak (asterisks ** at 125 kDa). Injected embryos were manually dechorionated and deyolked. Proteins were analyzed on 8% SDS-PAGE gels. Blots were blocked in 4% non-fat milk or 5% BSA and probed with antibody in 3% BSA. FAK C-20 (1:1000 dilution; sc-558) and FAK (1:200 dilution; Invitrogen AHO0502). GAPDH was used as a loading control (1:25,000 dilution, ab22555). Blots were visualized using Enhanced Chemiluminscence. (D) Representative brightfield images and quantification of *fak* morphant embryo rescue experiments. Control MO or *fak* MO was co-injected with mGFP, wild-type human *FAK,* or human FAK^{Y397E} mRNA. Each condition had equal total amounts of mRNA injected. Embryos were analyzed at prim-6 for MHB defects. Each embryo was scored as having a normal, mild, or severe phenotype. Mild phenotypes are represented in D. Control MO + mGFP (*n=38*), control MO + *wt hFAK* (*n=42*), *fak* MO + mGFP (*n=55*), *fak* MO + *hFAK* (*n=63*), control MO + $hFAK^{Y397E}$ (*n=51*), *fak* MO + $hFAK^{Y397E}$ (*n=54*). Anterior is to the left in all images. Arrowheads indicate MHBC.

Biology Open • Supplementary information

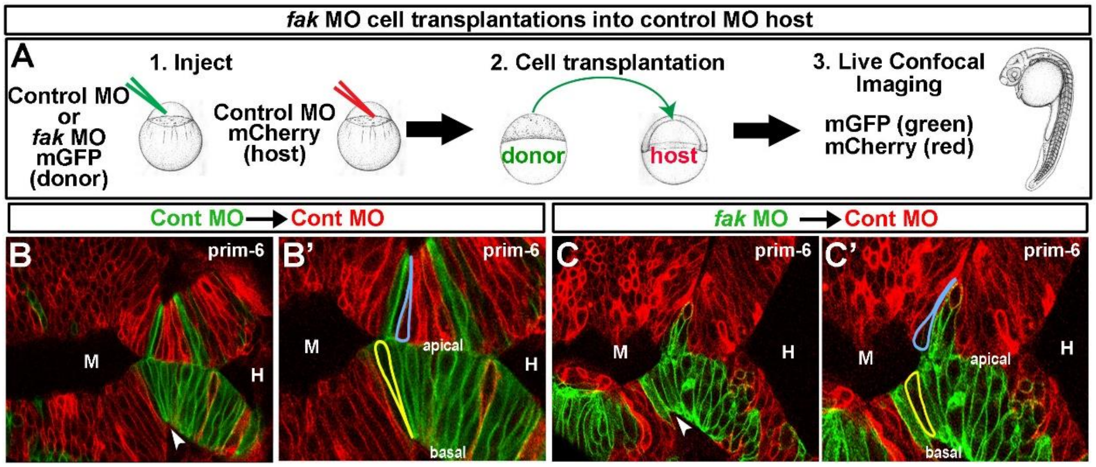

Fig. S4. Fak is required at the MHBC for basal constriction. Schematic for transplant procedure. 1. One-cell stage wild-type embryos were co-injected with mGFP (donor) or mCherry (host) and control or *fak* MO. 2. Cells from donors (sphere stage) were transplanted into hosts (shield stage). Transplanted cells were targeted to the presumptive MHB region (Woo and Fraser, 1995). 3. Embryos were incubated until prim-6 then imaged with live confocal microscopy. (E-F') Donor cells are outlined in yellow and host cells in blue. (B,B') Control donor and control host cells basally constricted normally when transplanted to the MHBC, (*n*=6). (C,C') Cells from *fak* morphant donors transplanted into control hosts failed to undergo basal constriction at the MHBC, (*n*=4). Basal constriction occurred normally in control host cells, even when immediately adjacent to *fak* morphant donor cells.

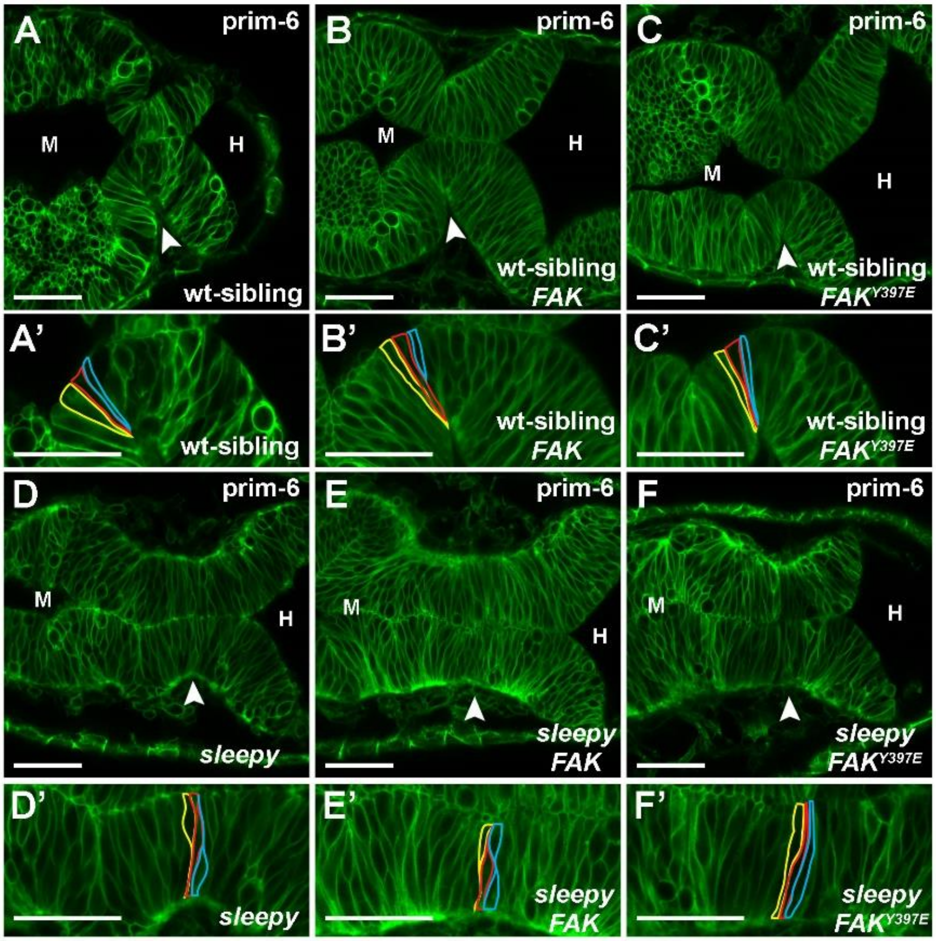

Fig. S5. Expression of human FAK does not rescue the laminin mutant basal constriction phenotype. (A-F') Live confocal images showing the MHB region of prim-6 embryos injected with mGFP (A,D), mGFP + wt *FAK* mRNA (B,E), mGFP + *FAK-Y397E* (C,F). (A-C') *sleepy* (*sly*^{m86}) (Schier et al., 1996) heterozygous sibling or wild-type sibling embryos showing normal basal constriction at the MHBC. (D-F') *sleepy* mutants showing defects in basal constriction both without and with the co-injection of human wild-type *FAK* and human phosphomimetic *FAK*^{Y397E} mRNA. Representative images from 3 independent experiments with n>3 for each condition. (A'-F') Magnifications of the neuroepithelium shown in A-F with individual cells outlined at the MHBC. Arrowheads indicate MHBC. M, midbrain. H, hindbrain. Scale bars: 35 μm.
